# RETRACTION: Surgical Site Infection Following Traumatic Orthopaedic Surgeries in Geriatric Patients: Incidence and Prognostic Risk Factors

**DOI:** 10.1111/iwj.70643

**Published:** 2025-04-23

## Abstract

**RETRACTION:** LeJ. , DongZ. , LiangJ. , ZhangK. , LiY. , ChengM. , and ZhaoZ. , “Surgical Site Infection Following Traumatic Orthopaedic Surgeries in Geriatric Patients: Incidence and Prognostic Risk Factors,” International Wound Journal 17, no. 1 (2020): 206–213, 10.1111/iwj.13258.31730274 PMC7949393

The above article, published online on 15 November 2019, in Wiley Online Library (http://onlinelibrary.wiley.com/), has been retracted by agreement between the journal Editor in Chief, Professor Keith Harding; and John Wiley & Sons Ltd. Following an investigation by the publisher, all parties have concluded that this article was accepted solely on the basis of a compromised peer review process. The editors have therefore decided to retract the article. The authors did not respond to our notice regarding the retraction.

**RETRACTION:** J. Le , Z. Dong , J. Liang , K. Zhang , Y. Li , M. Cheng , and Z. Zhao , “Surgical Site Infection Following Traumatic Orthopaedic Surgeries in Geriatric Patients: Incidence and Prognostic Risk Factors,” International Wound Journal 17, no. 1 (2020): 206–213, 10.1111/iwj.13258.31730274 PMC7949393

The above article, published online on 15 November 2019, in Wiley Online Library (http://onlinelibrary.wiley.com/), has been retracted by agreement between the journal Editor in Chief, Professor Keith Harding; and John Wiley & Sons Ltd. Following an investigation by the publisher, all parties have concluded that this article was accepted solely on the basis of a compromised peer review process. The editors have therefore decided to retract the article. The authors did not respond to our notice regarding the retraction.

